# Smartphone Sensor Data for Identifying and Monitoring Symptoms of Mood Disorders: A Longitudinal Observational Study

**DOI:** 10.2196/35549

**Published:** 2022-05-04

**Authors:** Taylor A Braund, May The Zin, Tjeerd W Boonstra, Quincy J J Wong, Mark E Larsen, Helen Christensen, Gabriel Tillman, Bridianne O’Dea

**Affiliations:** 1 Black Dog Institute University of New South Wales Sydney Australia; 2 Faculty of Medicine and Health University of New South Wales Sydney Australia; 3 Faculty of Psychology and Neuroscience Maastricht University Maastricht Netherlands; 4 School of Psychology Western Sydney University Sydney Australia; 5 School of Science, Psychology and Sport Federation University Ballarat Australia

**Keywords:** depression, bipolar disorder, sensors, mobile app, circadian rhythm, mobile phone

## Abstract

**Background:**

Mood disorders are burdensome illnesses that often go undetected and untreated. Sensor technologies within smartphones may provide an opportunity for identifying the early changes in circadian rhythm and social support/connectedness that signify the onset of a depressive or manic episode.

**Objective:**

Using smartphone sensor data, this study investigated the relationship between circadian rhythm, which was determined by GPS data, and symptoms of mental health among a clinical sample of adults diagnosed with major depressive disorder or bipolar disorder.

**Methods:**

A total of 121 participants were recruited from a clinical setting to take part in a 10-week observational study. Self-report questionnaires for mental health outcomes, social support, social connectedness, and quality of life were assessed at 6 time points throughout the study period. Participants consented to passively sharing their smartphone GPS data for the duration of the study. Circadian rhythm (ie, regularity of location changes in a 24-hour rhythm) was extracted from GPS mobility patterns at baseline.

**Results:**

Although we found no association between circadian rhythm and mental health functioning at baseline, there was a positive association between circadian rhythm and the size of participants’ social support networks at baseline (*r*=0.22; *P=*.03; *R^2^*=0.049). In participants with bipolar disorder, circadian rhythm was associated with a change in anxiety from baseline; a higher circadian rhythm was associated with an increase in anxiety and a lower circadian rhythm was associated with a decrease in anxiety at time point 5.

**Conclusions:**

Circadian rhythm, which was extracted from smartphone GPS data, was associated with social support and predicted changes in anxiety in a clinical sample of adults with mood disorders. Larger studies are required for further validations. However, smartphone sensing may have the potential to monitor early symptoms of mood disorders.

## Introduction

### Background

Circadian rhythm is an endogenous mammalian process that helps to regulate an individual’s activity over 24-hour cycles [1]. The temporal organization of the circadian rhythm is important for balancing physiological functions of body temperature and hormones [2] as well as behavioral schedules such as daily activities, sleep-wake patterns [3], and how individuals interact with each other in their social networks [4]. As such, its disruption can have substantial impacts on the health of individuals.

Circadian rhythm irregularities have been found to be closely related to the onset and clinical manifestations of mood disorders [5] such as major depressive disorder (MDD) and bipolar disorder (BD). Many studies investigating the relationship between circadian rhythm and mental health symptoms have used behavior questionnaires and self-reports of sleep-wake activity [6-11]. With different rhythm measures, studies have found correlations between circadian rhythm irregularity and greater symptoms of depression [12,13] and anxiety [13], poorer quality of life [12], and lower life satisfaction [13] and social support [14]. Moreover, disruptions in circadian rhythms differently affect episodes of mania and depression [15,16]. For example, melatonin—the hormone responsible for sustaining the sleep-wake cycle—has been found to be increased during mania and decreased during depression [16]. However, past studies are limited because of the methodological issues associated with low adherence rates for reporting and biases because of memory recall and subjectivity. Digital devices may provide a more nuanced identification and detection of circadian rhythm and present a clinical advantage for monitoring and managing mood disorders.

With advances in technology, mobile and wearable devices can now track specific behaviors and interactions [17], allowing researchers to address some of the drawbacks of traditional methods. More recently, modern smartphones equipped with powerful contextual sensors have made it feasible to capture participants’ symptoms with less burden [18]. Passive sensing through smartphones has enabled the gathering of information by operating in the background without the need for any input from the user [19] and without interrupting the user’s habitual routine. One of the commonly used sensors to study mood-related symptoms is a GPS, which maps a participant’s location [20-23]. For example, lesser mobility was found to be correlated with higher levels of mania [20] and depressive symptoms [20,21], with the relationships varying in strength depending on the timing throughout the week [22]. In addition, people with higher depressive scores showed decreased circadian rhythm, clustered around locations for longer periods, and had more disruptions to movement and variations in locations visited [23]. Other smartphone sensors included Bluetooth scans to estimate an individual’s social network based on proximity to other individuals’ mobile devices [18], device activity to denote social activity and predict communication frequencies [20], and accelerometers to infer physical and social activity [20,24], all of which may be used as proxies for behavioral markers of mood disorders. Given the ubiquity of smartphones today, this nonintrusive and objective method allows large-scale and population-level applications [25], which could help identify mood disorders early in the course of illness and provide a therapeutic tool through behavioral modification [26].

These past studies show promising findings, but they are hampered by significant limitations, including the lack of clinical participants [23] and small sample sizes [20,21,24,27]. Although some studies assessed participants through well-validated self-reported questionnaires during enrollment, most studies did not use a clinical interview conducted by a qualified clinician at baseline to determine diagnosis [18,21,22]. Even when the diagnoses were well established, the small sample sizes of most studies [20,21,24,27] limited statistical evidence and generalization. Furthermore, many studies used researcher-administered mobile phones rather than participants’ own smartphones [20,22-24,27], which may account for low adherence of completed self-assessments and missing data because participants reported forgetting to use the phone or carry it with them. Although initial studies have provided preliminary evidence for the link between smartphone measurements and clinical symptoms of mood disorders, additional research is required to provide stronger evidence for the relationship between sensor data and depressive symptoms.

### Objectives

The aim of this study was to determine the relationship between circadian rhythm in GPS data and symptoms of mental health measures across 6 time points among a clinical sample of adults diagnosed with MDD or BD. It was hypothesized that higher baseline circadian rhythm would be negatively associated with baseline symptoms of depression, mania, and anxiety [23] and positively associated with social support and quality of life [12-14]. Furthermore, symptoms of depression, anxiety, and mania would fluctuate over time in both disorders [28-30]. Finally, baseline circadian rhythm would predict change in mental health symptoms over time [22], with patients with BD showing a greater sensitivity to change [15,16].

## Methods

### Design

A 10-week prospective longitudinal design was used. Mental health measures were administered at baseline, on a fortnightly basis, and end point (10 weeks after baseline).

### Participants

Participants were recruited from the Black Dog Institute Depression and Bipolar Clinic, an in-person psychiatric diagnostic and treatment service for adults located in Sydney, New South Wales, Australia (for the CONSORT [Consolidated Standards of Reporting Trials] chart, see Multimedia Appendix 1). Adults are referred to this service by their general practitioner. Clinic administrative staff approached potential participants after they had completed their in-person diagnostic assessment with the trained psychiatrist. Participants were invited to participate in the study if their psychiatric assessment revealed a diagnosis of MDD or BD according to the criteria in the Diagnostic and Statistical Manual of Mental Disorders, Fifth Edition [31], were aged between 18 and 65 years, fluent in English, and owned a compatible smartphone (Android version≥4.4 or iOS version≥8). Participants were excluded if they had severe mood disturbance or suicidality where intervention was required as assessed by clinic staff, had current or past psychosis, or lacked access to a suitable smartphone for the duration of the study. Clinic administrative staff registered eligible participants through the study website and emailed the consent form to them. Participation was voluntary, and no reimbursement, financial incentive, or reward was provided.

### Ethics Approval

Ethics approval was obtained from the University of New South Wales Human Research Ethics Committee (HC17252).

### Sample Size

A sample size of 76 was required to detect a weak association (*r*=–0.3) between circadian rhythm and mental health functioning, with statistical power level 0.8 and *α*=.05. However, considering participant dropout and loss of smartphone data for some participants, the target was set at 200, with 100 participants for each diagnostic group.

### Procedure

After they provided consent, participants were instructed to install the study smartphone app Socialise [26] to complete the mental health measures and enable data collection (see Multimedia Appendix 2 for schedule). Participants were then instructed to use their phones normally for the study duration, with the app open in the background. Socialise was configured to automatically record GPS data and to prompt participants to complete the study questionnaires. Socialise conducted GPS data acquisition scans every 3, 4, 5, and 8 minutes.

### Measures

#### Circadian Rhythm

Circadian rhythm (also known as circadian movement [22] and quotidian movement [32]) was defined as the extent to which an individual’s sequence of locations followed a 24-hour rhythm [23]. This was determined at baseline based on changes in GPS location measured during the first 2 weeks of the study only when sufficient data were available. Least squares spectral analysis was performed to estimate the amount of energy that fell into 24-hour frequency bins [33]. Circadian rhythm was then calculated as the logarithm of the sum of energy for longitude and latitude (Multimedia Appendix 3 [22,23,33]). Hence, participants who regularly change their location at the same time each day will show a stronger 24-hour rhythm in their GPS data and have a higher circadian rhythm. Conversely, participants with irregular movement patterns (changing their location at different times each day) will have a lower circadian rhythm.

#### Depressive Symptoms

The Patient Health Questionnaire-9 (PHQ-9) [34] is a 9-item self-report questionnaire that measures the frequency of depressive symptoms during the past 2 weeks. Items are scored on a 4-point Likert scale of 0 (not at all) to 3 (nearly every day) and summed for a total score (range 0 to 27), with higher scores indicating greater depression. The total sum also corresponds to depression severity: none to minimal (0 to 4), mild (5 to 9), moderate (10 to 14), moderately severe (15 to 19), and severe (20 to 27). The PHQ-9 has good sensitivity (88%) and specificity (88%) for detecting likelihood of major depression using a cutoff of ≥10. Test-retest reliability in adults has been found to be acceptable (*r*=0.84), and internal consistency was strong (Cronbach *α*=.89) [34]. The PHQ-9 was administered at baseline, then on a fortnightly basis, with 6 surveys administered in the study period.

#### Anxiety Symptoms

The Generalized Anxiety Disorder Scale (GAD-7) [35] is a 7-item self-report questionnaire used to assess anxiety symptoms during the past 2 weeks. Each item is scored on a 4-point Likert scale of 0 (not at all) to 3 (nearly every day). Items are added together for a total score (range 0 to 21), with higher scores indicating greater anxiety. The total sum also corresponds to anxiety severity: minimal (0 to 4), mild (5 to 9), moderate (10 to 14), and severe (15 to 21). The GAD-7 has good sensitivity (89%) and specificity (82%) for detecting likelihood of anxiety disorder using a cutoff of ≥10 [36]. It has been found to demonstrate good internal consistency (Cronbach *α*=.92) and test-retest reliability (*r*=0.83) [35]. The GAD-7 was administered at baseline, then on a fortnightly basis, and at the end point, with 6 surveys administered in the study period.

#### Mania Symptoms

The 5-item Altman Self-Rating Mania Scale (ASRM) is a self-report questionnaire used to evaluate the presence and severity of manic symptoms in the past week [37]. There are 5 groups of statements, each corresponding to scores of 0 to 4, with 0 being unchanged behavior and 4 being frequent manic thoughts or behavior. The item scores are summed to give a total score (range 0 to 20), with a score of ≥6 indicating mania and higher scores indicating greater severity of symptoms. This scale has good sensitivity (87.3%) and specificity (85.5%) in adults [37]. The ASRM was administered at baseline, then on a fortnightly basis, and at end point, with 6 surveys administered in the study period.

#### Social Connectedness

The degree to which participants felt connected to others was measured using the 20-item Social Connectedness Scale-Revised (SCS-R) [38]. The scale consists of 20 items, with 10 positively worded questions and 10 negatively worded questions. It assesses participants’ experience with social inclusion, safety in their communities, and relationships with friends and families. The items are rated on a scale of *1* (strongly disagree) to *6* (strongly agree). Negatively worded items are reverse scored and summed with the scores of the remaining items to obtain a total score (range 20 to 120). Higher scores indicate a greater social connectedness to others. The SCS-R is a highly reliable measure with an internal consistency of Cronbach *α*=.92 [38]. The SCS-R was administered at baseline and end point.

#### Social Support

The 12-item Social Support Questionnaire (SSQ) [39] was used to measure 2 constructs of social support (size of support network and support satisfaction). The first 6 items evaluate the number of people that the participant feels could provide social support in the situations presented. Participants are asked to list initials and the type of relationship for each individual. The size of an individual’s support network or the SSQ number score (range 0 to 9) is then calculated by adding the number of individuals listed across all the items and dividing by 6. Higher SSQ number scores indicate a larger and more diverse social support network. The second part of the questionnaire assesses participants’ satisfaction with the support they receive in each situation. Items are scored on a 6-point scale ranging from 1 (very dissatisfied) to 6 (very satisfied). Similarly, scores are summed and divided by 6 to obtain an SSQ satisfaction score (range 1 to 6). Higher SSQ satisfaction scores indicate greater satisfaction with the support received. The SSQ has demonstrated good test-retest reliability (*r*=0.83) and high internal consistency (Cronbach *α*=.97) [39]. The SSQ was administered at baseline and end point.

#### Quality of Life

The Satisfaction With Life Scale (SWLS) [40] was used to measure participants’ life satisfaction and subjective quality of life. The self-report measure consists of 5 items rated on a 7-point Likert scale ranging from 1 (strongly disagree) to 7 (strongly agree). The total sum of the items ranges from 5 to 35, with a score of 20 indicating a neutral score and lower scores indicating lower satisfaction with life. The total sum also corresponds to satisfaction level: extremely dissatisfied (5 to 9), dissatisfied (10 to 14), slightly below average in life satisfaction (15 to 19), neutral (20 to 24), highly satisfied (25 to 29), and extremely satisfied (30 to 35). The SWLS has good psychometric properties, including internal consistency with the coefficient Cronbach *α* ranging from .79 to .89 and test-retest reliability of *r*=0.84 over a 1-month period [41]. The SWLS was administered at baseline and end point.

### Data Collection

The Socialise app was configured to automatically record GPS data. The app conducted GPS data acquisition scans every 3, 4, 5, and 8 minutes. The app was also configured to automatically schedule the study questionnaires at fortnightly intervals and prompt participants to complete them. Using internet connectivity, the app transferred all participant data to the Black Dog Institute research platform, hosted on University of New South Wales servers. Data were then downloaded and analyzed by the research team. Because of an issue with app connectivity, an error in the final survey scheduling was detected. This resulted in the final survey being delivered at 9 weeks after baseline instead of 10 weeks.

### Statistical Analysis

We used 2-tailed *t* tests to examine differences between participants with MDD and those with BD in circadian rhythm and mental health outcomes, social support, social connectedness, and quality of life at baseline. Chi-square tests were used to test whether the proportion of participants meeting cutoff scores of mental health disorder diagnoses at baseline differed between participants with MDD and those with BD. Linear regression models were used to test whether baseline circadian rhythm was associated with baseline mental health, social support, social connectedness, or quality of life. Interaction terms between circadian rhythm and diagnostic group were first included to test whether the relationships differed between diagnostic groups.

Mixed linear models were used to test whether mental health functioning changed over time. Models included mental health, social support, or social connectedness as the dependent variable; participant as the random effect; and time point as a fixed effect. Interaction terms between the fixed effects of time point and diagnostic group were first included to test whether changes in mental health and social functioning differed between people with MDD and those with BD. Post hoc tests were performed using estimated marginal means, with the false discovery rates (FDRs) for all *P* values corrected to *q* values using the Benjamini and Hochberg [42] procedure.

Mixed linear models were also used to test whether circadian rhythm moderated changes in mental health or social support/connectedness over time. Models included mental health, social support, or social connectedness as the dependent variable; subject as the random effect; and time point as a fixed effect. Interaction terms between the fixed effects of time point and circadian rhythm were then used to test whether baseline circadian rhythm moderated changes in mental health and social functioning. Interaction terms among the fixed effects of time point, circadian rhythm, and diagnostic group were first included to test whether the moderating effects of baseline circadian rhythm on changes in mental health and social functioning differed between people with MDD and those with BD. Post hoc tests were performed using estimated marginal means, with the FDRs for all *P* values corrected to *q* values using the Benjamini and Hochberg [42] procedure.

All analyses were conducted using R software (version 3.5.1; The R Foundation for Statistical Computing) [43]. Mixed linear models were tested using the *lme4* package in R [44]. *P* values for mixed linear models were calculated using the *lmerTest* package in R [45]. Post hoc tests were performed using the *emmeans* package in R [46] (see Multimedia Appendix 4 for supplementary analyses).

## Results

### Participants

During the recruitment period, 219 adults attended the Black Dog Institute clinic for a psychiatric assessment (Multimedia Appendix 1), of whom 162 (74%) were deemed eligible for the study by clinic administrative staff and were invited to participate. Of these 162 participants, 149 (92%) consented, downloaded the Socialise app, and completed the baseline mental health assessment; however, of these 149 participants, 3 (2%) withdrew shortly after completion of baseline assessment and 25 (16.8%) did not have sufficient GPS data, leaving a final sample that consisted of 121 (83.2%) participants, with attrition for the study questionnaires outlined in Multimedia Appendix 2.

### Demographic and Clinical Characteristics

Table 1 outlines the baseline demographic and clinical characteristics of the final sample (n=121). The mean age of participants was 41.4 (SD 13.6; range 18 to 70) years; 65.3% (79/121) were women; and 66.1% (80/121) used iPhones. People with MDD had significantly higher anxiety (*t*_119_=–2.47; *P*=.02; Cohen *d*=–0.47, 95% CI –0.85 to –0.09) and likely cases of anxiety (*χ*^2^_1_=6.1; *P*=.01; *φ*=0.24, 95% CI 0.06-0.42), lower mania (*t*_119_=2.74; *P*=.007; Cohen *d*=0.52, 95% CI 0.14-0.90), and a smaller social support network (*t*_119_=2.57; *P*=.01; Cohen *d*=0.49, 95% CI 0.11-0.87) than participants with BD. There was no significant relationship between diagnosis and likely cases of depression, *χ*^2^_1_=0.9; *P*=.35, or mania, *χ*^2^_1_=3.0; *P*=.08. No other differences in mental health or social support/connectedness were found.

**Table 1:** Baseline demographic and clinical characteristics of participants (N=121).

		Total sample	Depression (n=79)	Bipolar disorder (n=42)	*t* test (*df*)	Chi-square test (*df*)	*P* value
Age (years), mean (SD)		41.41 (13.62)	41.63 (13.94)	41.00 (13.16)	–0.24 (119)	N/A^a^	.81
Depressive symptoms (PHQ-9^b^), mean (SD)		11.75 (6.67)	12.51 (7.09)	10.33 (5.61)	–1.85 (119)	N/A	.07
Anxiety symptoms (GAD-7^c^), mean (SD)		8.21 (5.76)	9.14 (5.93)	6.48 (5.04)	–2.60 (119)	N/A	*.01* ^d^
Mania symptoms (ASRM^e^), mean (SD)		4.12 (3.06)	3.58 (2.63)	5.14 (3.56)	2.50 (119)	N/A	*.02*
Quality of life (SWLS^f^), mean (SD)		16.08 (7.51)	15.22 (7.43)	17.71 (7.49)	1.76 (119)	N/A	.08
Social connectedness (SCS-R^g^), mean (SD)		73.57 (4.25)	73.71 (4.51)	73.29 (3.99)	–0.21 (19)	N/A	.83
**Social support, mean (SD)**							
	SSQNS^h^	2.63 (1.60)	2.37 (1.38)	3.13 (1.86)	2.57 (119)	N/A	*.01*
	SSQSS^i^	4.31 (1.29)	4.25 (1.24)	4.41 (1.39)	0.65 (119)	N/A	.52
Circadian rhythm, mean (SD)		–5.46 (3.58)	–5.43 (3.71)	–5.50 (3.38)	–0.09 (94)	N/A	.93
Sex (female), n (%)		79 (65.3)	50 (63.3)	29 (69)	N/A	0.2 (1)	.67
**Likely clinical case, n (%)**							
	Depressive symptoms (PHQ-9 score≥10)	69 (57)	48 (60.8)	21 (50)	N/A	0.9 (1)	.35
	Anxiety symptoms (GAD-7 score≥10)	39 (32.2)	32 (40.5)	7 (16.7)	N/A	6.1 (1)	*.01*
	Mania symptoms (ASRM score≥6)	33 (27.3)	17 (21.5)	16 (38.1)	N/A	3.0 (1)	.08

^a^N/A: not applicable.

^b^PHQ-9: Patient Health Questionnaire-9.

^c^GAD-7: Generalized Anxiety Disorder Scale.

^d^Italicization indicates values that met the significance threshold (*P*<.05).

^e^ASRM: Altman Self-Rating Mania Scale.

^f^SWLS: Satisfaction With Life Scale.

^g^SCS-R: Social Connectedness Scale-Revised.

^h^SSQNS: Social Support Questionnaire number score.

^i^SSQSS: Social Support Questionnaire satisfaction score.

### Association Among Baseline Circadian Rhythm, Mental Health, and Social Support/Connectedness

**Figure 1:**
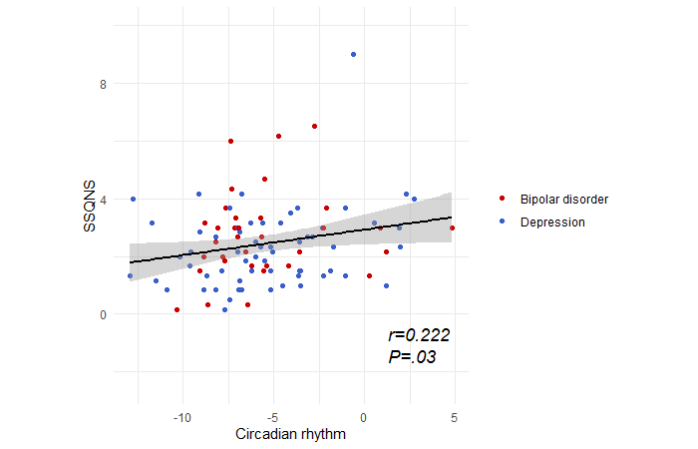
Association between circadian rhythm and the size of social support networks (Social Support Questionnaire number score [SSQNS]). Shading represents 95% CIs.

There were no significant interactions between circadian rhythm and diagnostic group in predicting mental health or social support/connectedness; therefore, data were pooled across diagnostic groups. At baseline, higher circadian rhythm was associated with a larger social support network (*β*=.088, 95% CI 0.009-0.168; *t*_94_=2.20; *P*=.03; Figure 1). Circadian rhythm was not significantly associated with any other measure of mental health or social support/connectedness.

### Changes in Mental Health and Social Support/Connectedness Over Time

**Figure 2:**
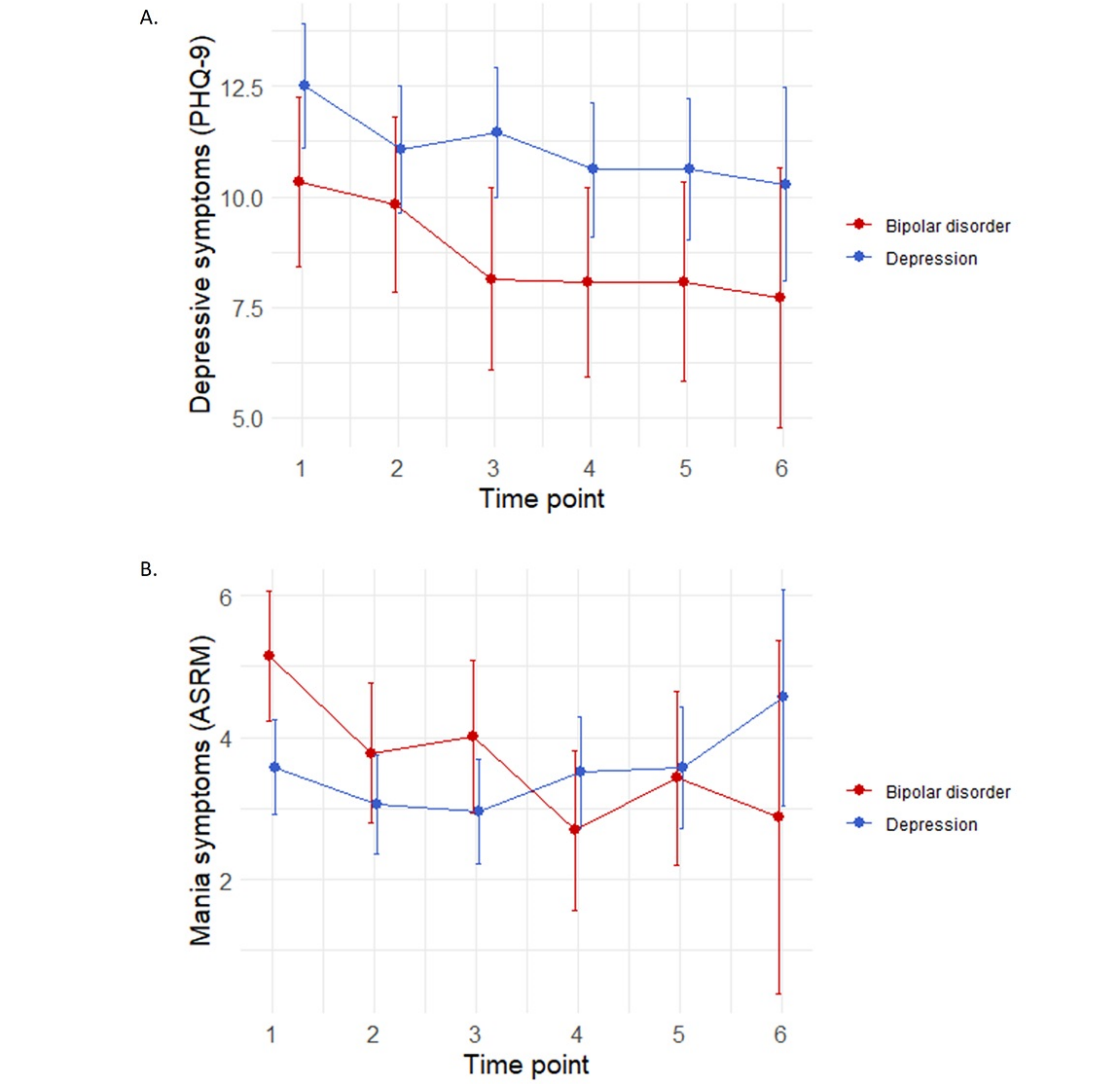
(A) Depressive symptoms (Patient Health Questionnaire-9 [PHQ-9]) and (B) mania symptoms (Altman Self-Rating Mania Scale [ASRM]) at each study time point. Error bars represent 95% CIs.

There was no interaction between diagnosis and time point in predicting PHQ-9 scores (*F*_5,359_=0.94; *P*=.46); therefore, diagnostic groups were pooled together for analysis. There was a significant main effect of time point in predicting PHQ-9 scores (*F*_5,363_=4.92; *P*<.001). Post hoc tests found that PHQ-9 scores decreased from baseline to time point 2 (*t*_367_=2.58; *q*=.03; Cohen *d*=0.35, 95% CI 0.08-0.62), time point 3 (*t*_370_=3.05; *q*=.01; Cohen *d*=0.44, 95% CI 0.15-0.72), time point 4 (*t*_371_=4.09; *q*<.001; Cohen *d*=0.62, 95% CI 0.32-0.92), time point 5 (*t*_370_=3.77; *q*=.002; Cohen *d*=0.61, 95% CI 0.29-0.94), and time point 6 (*t*_368_=2.95; *q*=.01; Cohen *d*=0.73, 95% CI 0.24-1.23; Figure 2A). There were no other significant differences among the time points. There were no other significant interactions between diagnosis and time points or main effects for the time points for other measures of mental health or social support/connectedness.

There was a significant interaction between time point and diagnosis in predicting mania on the ASRM (*F*_5,360_=2.96; *P*=.01). Post hoc tests found that ASRM scores in people with BD decreased from baseline to time point 4 (*t*_375_=4.06; *q*=.004; Cohen *d*=1.06, 95% CI 0.55-1.59]; Figure 2B). Compared with baseline ASRM scores in people with BD, ASRM scores in people with MDD were also lower at time point 2 (*t*_293_=3.60; *q*=.01; Cohen *d*=0.91, 95% CI 0.41-1.42) and time point 3 (*t*_305_=3.63; *q*=.01; Cohen *d*=0.95, 95% CI 0.43-1.47).

### Circadian Rhythm Predicting Change in Mental Health or Social Support/Connectedness Over Time

**Figure 3:**
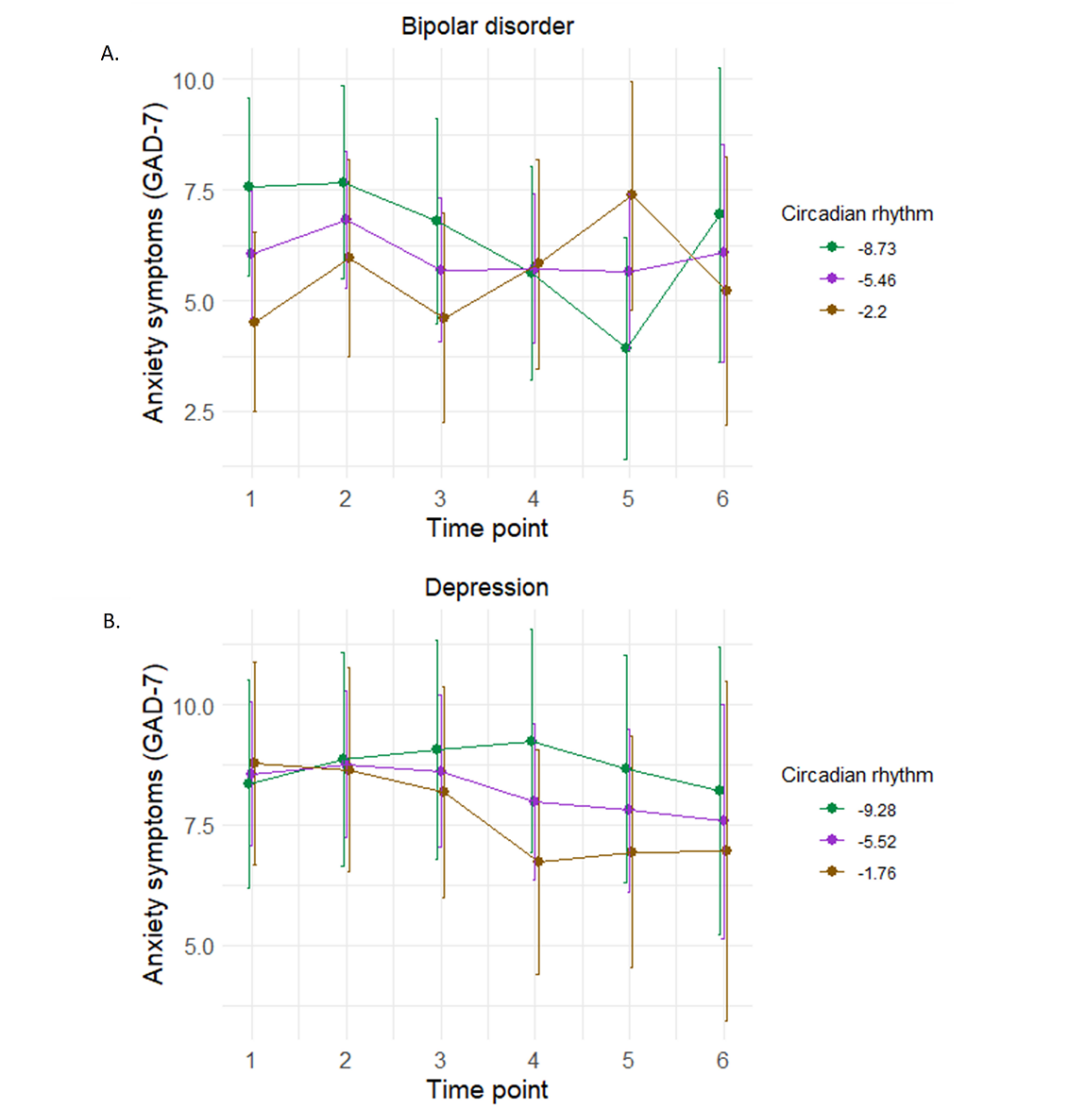
Baseline circadian rhythm moderating change in anxiety (Generalized Anxiety Disorder Scale [GAD-7]) at each study time point in (A) bipolar disorder and (B) depression. Following the convention suggested by Aiken and West [47], we used the mean value of the moderator (ie, circadian rhythm) as well as 1 SD below and above the mean value of the moderator to plot the effect of circadian rhythm on change in anxiety across time points. Error bars represent 95% CIs.

A significant interaction was found among time point, baseline circadian rhythm, and diagnoses (*F*_5,275_=3.65; *P*=.003) in predicting GAD-7 scores. To further explore the 3-way interaction, MDD and BD were analyzed separately. A significant interaction between time point and circadian rhythm was found for people with BD only (*F*_5,91_=3.25; *P*=.009; Figure 3A [47]). Post hoc tests found that anxiety in people with circadian rhythm scores approximately 1 SD below the mean (ie, –8.73) decreased between time points 1 and 5 (*t*_94.6_=3.0; *P*=.003; *q*=.02; Cohen *d*=1.34, 95% CI 0.44-2.25). Conversely, anxiety in people with circadian rhythm scores 1 SD above the mean (ie, –2.20) significantly increased between time points 1 and 5; yet, this did not survive FDR correction (*t*_94.6_=–2.32; *P*=.02; *q*=.11; Cohen *d*=–1.05, 95% CI –1.98 to –0.13). No interaction was found between time points and circadian rhythm for people with MDD (Figure 3B [47]). No significant interactions were found among time point, circadian rhythm, and diagnosis category or time point and circadian rhythm for any other measures of mental health (Multimedia Appendix 5) or social support/connectedness.

## Discussion

### Principal Findings

This study investigated the association among circadian rhythm, mental health symptoms, and social support/connectedness in adults with MDD or BD. Although we found no significant association between circadian rhythm and mental health scores, we found a positive association between circadian rhythm and social support at baseline. Both clinical groups showed a decrease in depressive symptoms over time; yet, only those with BD showed a decrease in mania symptoms. Finally, we found that circadian rhythm at baseline moderated the change in anxiety symptoms in people with BD only, where higher baseline circadian rhythm scores were associated with increased anxiety and lower baseline circadian rhythm scores were associated with decreased anxiety at follow-up.

Circadian rhythm was not associated with mental health symptoms; yet, it was positively associated with the size of participants’ social support networks. This association aligns with the study by Margraf et al [13], who found that greater social rhythm regularity was associated with better life satisfaction in healthy individuals. Greater circadian rhythm reflects individuals’ increased movement around various locations, suggesting that they may be more socially interactive. Social interaction also plays an important role in well-being [48], which could lead to improved mental health. Although previous studies have established relationships between circadian rhythm and other measures of mental health functioning, the samples are generally made up of only participants with BD [12], healthy controls and participants with MDD [14], healthy individuals, or healthy adolescents [49]. Moreover, although Saeb et al [23] found a strong negative association (*r*=–0.63) between circadian rhythm and PHQ-9 scores, their limited analyzable sample size (n=28) may have overestimated the association. In this study, we found a nonsignificant association using a larger sample size (n=121), with the coefficient reported by Saeb et al [23] not found within our 95% CIs (*r*=–0.12, 95% CI –0.32 to 0.08). This finding is also in line with the follow-up study by Saeb et al [22], who found a nonsignificant association between circadian movement and PHQ-9 scores at baseline and a weaker correlation coefficient (*r*=–0.34, 95% CI –0.339 to 0.341). Taken together, our findings suggest that only social support may be associated with circadian rhythm at baseline, whereas little evidence is found for the association between circadian rhythm and mental health functioning.

Although both clinical groups showed a decrease in depressive symptoms over time, only those with BD showed a decrease in mania symptoms. These findings are in line with previous mental health studies that report fluctuations in mental health symptoms over time [28-30]. Moreover, given that BD is uniquely characterized by manic features [31,50], variation in these symptoms would be expected among people with BD. However, given the overall trajectories of symptom profiles, the study duration may not have been adequate to accurately capture the true symptom variability. This pattern of symptoms implies that future studies would benefit from longer durations and more incentives to increase study completion.

Circadian rhythm moderated the change in anxiety symptoms in people with BD only, where higher baseline circadian rhythm was associated with increased anxiety and lower baseline circadian rhythm was associated with decreased anxiety between baseline and time point 5. People with BD had significantly lower baseline anxiety symptoms and likely cases of anxiety than people with MDD, suggesting differing anxiety profiles between the diagnostic groups. Furthermore, increased anxiety in those with higher baseline circadian rhythm may reflect an initial decline in functioning that corresponds with poor mental health. Similarly, if circadian rhythm is lower during a period of poor mental health, it may then predict improvements in anxiety at future time points as one recovers. However, these effects seem to manifest dynamically in the symptom cycle because the effect disappears by the final time point. Together, these findings suggest that circadian rhythm may be a marker for anxiety symptom trajectories in people with BD that can be monitored for targeted treatment and intervention.

This study includes several limitations. Although the recruitment of clinically diagnosed patients is the strength of the study, the lack of healthy controls limits generalizability from clinical to nonclinical samples. Despite being recruited through a clinical setting, our participants reported low levels of symptoms, suggesting that they may be in a relatively stable symptomatic period. As such, future research should explore participants across different stages of the course of illness in longitudinal studies. Although GPS data offers an inexpensive and accessible data source for the tracking of circadian rhythm, the direct relationship to biological measures of circadian rhythm remains unclear and requires further investigation. Problems with data uploads were generally because of lost internet connection and disabled mobile phone settings (ie, GPS, internet, notifications, and data), which limited the analysis of GPS data to baseline only. Moreover, although enough participants expressed interest in terms of our target sample size, there was a considerable amount of participant dropout and missing data. Specifically, the number of participants with BD (n=42) was lower than those with MDD (n=76) and did not meet the target sample size (n=76). Future research may benefit from developing study apps that send push notifications to remind participants to enable certain settings and complete the required questionnaires.

### Conclusions

In conclusion, our results suggest that circadian rhythm extracted from smartphone GPS data was associated with social support and change in anxiety in a clinical sample of adults with mood disorders. However, little evidence was found for the association between circadian rhythm and mental health functioning. Larger future studies examining a wider variety of biomarkers and sensor features for a longer duration of time are warranted. However, with the ubiquity of smartphones, there is an encouraging potential to shift the nature of identifying mood disorders. Contextual features translated directly from smartphone data may potentially detect mood disorders earlier and more easily.

## Appendix

Multimedia Appendix 1 Recruitment flow of the study.

Multimedia Appendix 2 Schedule and completion of the study questionnaires.

Multimedia Appendix 3 Circadian rhythm.

Multimedia Appendix 4 Supplementary analyses.

Multimedia Appendix 5 Nonsignificant moderating effect of baseline circadian rhythm change in (A) depression severity (Patient Health Questionnaire-9) and (B) mania (Altman Self-Rating Mania Scale) across time points. Error bars represent 95% CIs.

## Abbreviations

ASRM: Altman Self-Rating Mania Scale
BD: bipolar disorder
CONSORT: Consolidated Standards of Reporting Trials
FDR: false discovery rate
GAD-7: Generalized Anxiety Disorder Scale
MDD: major depressive disorder
PHQ-9: Patient Health Questionnaire-9
SCS-R: Social Connectedness Scale-Revised
SSQ: Social Support Questionnaire
SWLS: Satisfaction With Life Scale
