# Supplementary material for: Smartphone Sensor Data for Identifying and Monitoring Symptoms of Mood Disorders: A Longitudinal Observational Study
Source: JMIR Ment Health. 2022 May 4;9(5):e35549. doi: 10.2196/35549 (PMC9118091; doi:10.2196/35549)
Supplement: Multimedia Appendix 1 [file mental_v9i5e35549_app1.docx]

Multimedia Appendix 1. Recruitment flow for the study

**Screening**

# Screening

Attended the clinic for an in-person psychiatric assessment (*n* = 219)

Excluded (*n* = 57)

- No MDD or BP DSM diagnosis (n = 57)

**Enrollment**

Downloaded Socialise study app and provided informed consent (*n* = 162)

# Enrollment

**Study Period**

Smartphone Data Collection

Circadian rhythm extracted from GPS and completed mental health questionnaires (PHQ-9, GAD-7, ASRM, SCS-R, SSQ, SWLQ)

#

Analysed (*n* = 121)

Excluded (*n* = 41)

- Withdrawal (*n* = 3)
- Baseline assessment not completed (*n* = 13)
- Incomplete GPS data (*n* = 25)

**Analysis**

# Analysis

# Study Period
