# Supplementary material for: Smartphone Sensor Data for Identifying and Monitoring Symptoms of Mood Disorders: A Longitudinal Observational Study
Source: JMIR Ment Health. 2022 May 4;9(5):e35549. doi: 10.2196/35549 (PMC9118091; doi:10.2196/35549)
Supplement: Multimedia Appendix 2 [file mental_v9i5e35549_app2.docx]

Multimedia Appendix 2. Schedule and completion of the study questionnaires.

|  | **Timepoint (Questionnaire number)** | | | | | |
| --- | --- | --- | --- | --- | --- | --- |
|  | **Baseline**  **(1)** | **Day 14**  **(2)** | **Day 28**  **(3)** | **Day 42**  **(4)** | **Day 56**  **(5)** | **Final endpoint (6)** |
| Depressive symptoms (PHQ-9) | 121 | 107 | 93 | 78 | 63 | 22 |
| Anxiety symptoms (GAD-7) | 121 | 107 | 90 | 76 | 62 | 19 |
| Mania symptoms (ASRM) | 121 | 106 | 90 | 77 | 62 | 15 |
| Social connectedness (SCS-R) | 121 | - | - | - | - | 21 |
| Quality of life (SWLQ) | 121 | - | - | - | - | 22 |
| Social support - SSQS | 121 | - | - | - | - | 20 |
| Social support - SSQN | 121 | - | - | - | - | 20 |
