# Supplementary material for: Smartphone Sensor Data for Identifying and Monitoring Symptoms of Mood Disorders: A Longitudinal Observational Study
Source: JMIR Ment Health. 2022 May 4;9(5):e35549. doi: 10.2196/35549 (PMC9118091; doi:10.2196/35549)
Supplement: Multimedia Appendix 3 [file mental_v9i5e35549_app3.docx]

Multimedia Appendix 3. Circadian rhythm.

In line with Saeb, Lattie [1], [2], circadian rhythm was calculated using the following method. First, we used the least-squares spectral analysis [3] to obtain the spectrum of the GPS signals. Then, we calculated the amount of energy that fell into the frequency bins within a 24 ± 0.5 h period, in the following way:


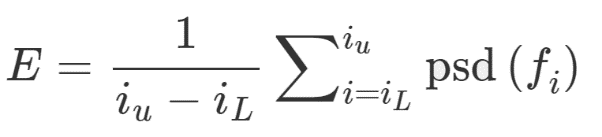


where psd(*f*i) denotes the power spectral density at frequency bin *fi*, and *iL* and *iU* represent the lower and the upper bounds of the frequency range of interest, corresponding to 24.5 and 23.5 h periods respectively. We calculated *E* separately for longitude and latitude, and obtained the total circadian movement as:

**
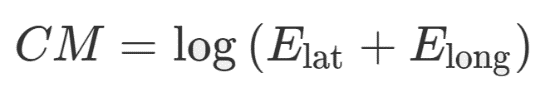
**

References

1. Saeb, S., et al., *The relationship between mobile phone location sensor data and depressive symptom severity.* PeerJ, 2016. **4**: p. e2537.

2. Saeb, S., et al., *Mobile Phone Sensor Correlates of Depressive Symptom Severity in Daily-Life Behavior: An Exploratory Study.* J Med Internet Res, 2015. **17**(7): p. e175.

3. Press, W.H., et al., *Numerical recipes 3rd edition: the art of scientific computing*. 2007: Cambridge university press.
