# Supplementary material for: Smartphone Sensor Data for Identifying and Monitoring Symptoms of Mood Disorders: A Longitudinal Observational Study
Source: JMIR Ment Health. 2022 May 4;9(5):e35549. doi: 10.2196/35549 (PMC9118091; doi:10.2196/35549)
Supplement: Multimedia Appendix 4 [file mental_v9i5e35549_app4.docx]

Multimedia Appendix 4. Supplementary analysis.

Participants were sent the study questionnaires at the same time of day but did not complete the questionnaires on the same day of the week or the same time of day due to the recruitment procedure. However, all baseline assessments were completed on weekdays and generally distributed between 9am and 5pm.

Distribution of day of week and time of data collection

Frequency

|  | Monday | Tuesday | Wednesday | Thursday | Friday |
| --- | --- | --- | --- | --- | --- |
| *n* | 1 | 2 | 31 | 38 | 49 |


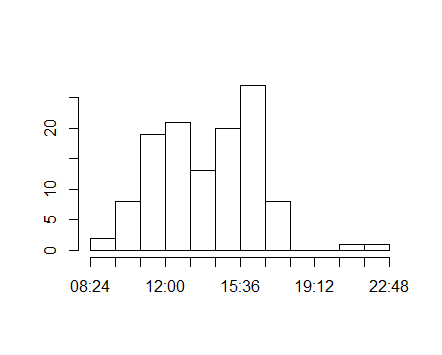


Time

Furthermore, including date and time as fixed effect covariates in longitudinal models did not change any results reported in the manuscript (see below for comparisons).

|  | **Without day and time covariates** | | **With day and time covariates** | |
| --- | --- | --- | --- | --- |
| **Results reported in manuscript** | *F* | *p* | *F* | *P* |
| Interaction between timepoint and diagnosis in predicting ASMR. | 2.96 | .012 | 2.98 | 0.012 |
| Timepoint in predicting PHQ-9. | 4.92 | < .001 | 4.76 | < .001 |
| Interaction between timepoint, baseline circadian rhythm, and diagnoses in predicting GAD-7. | 3.65 | .003 | 3.63 | .003 |
| Interaction between timepoint and baseline circadian rhythm in predicting GAD-7 for people with BP only. | 3.25 | .009 | 3.17 | .011 |
