# Supplementary material for: Smartphone Sensor Data for Identifying and Monitoring Symptoms of Mood Disorders: A Longitudinal Observational Study
Source: JMIR Ment Health. 2022 May 4;9(5):e35549. doi: 10.2196/35549 (PMC9118091; doi:10.2196/35549)
Supplement: Multimedia Appendix 5 [file mental_v9i5e35549_app5.docx]

Multimedia Appendix 5. Nonsignificant moderating effect of baseline circadian rhythm change in (A) depression severity (Patient Health Questionnaire-9) and (B) mania (Altman Self-Rating Mania Scale) across time points. Error bars represent 95% CIs.

a)

b)


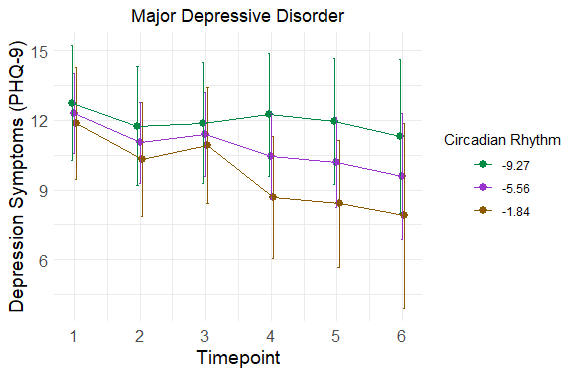

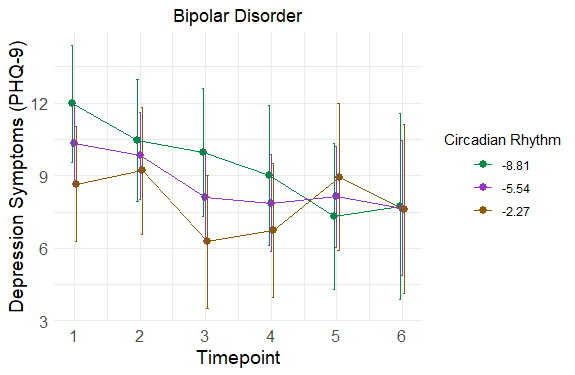


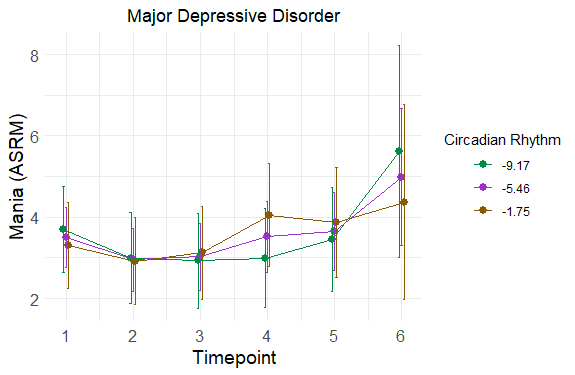

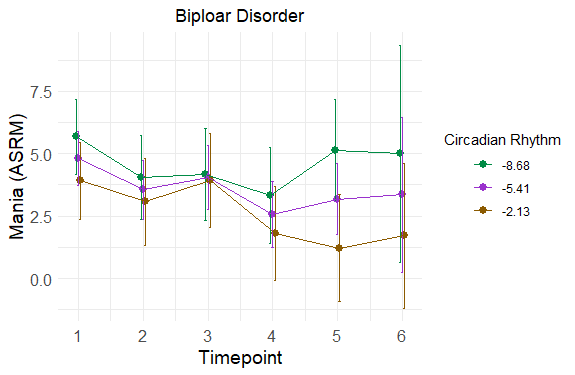


Note: Error bars represent 95% confidence intervals.
